# Drought induces opposite changes in organ carbon and soil organic carbon to increase resistance on moso bamboo

**DOI:** 10.3389/fpls.2024.1474671

**Published:** 2024-11-26

**Authors:** Xiaogai Ge, Yilian Mao, Benzhi Zhou, Xiaoming Wang, Mai–He Li

**Affiliations:** ^1^ Research Institute of Subtropical Forestry, Chinese Academy of Forestry, Hangzhou, Zhejiang, China; ^2^ Qianjiangyuan Forest Ecosystem Research Station, National Forestry and Grassland Administration, Hangzhou, Zhejiang, China; ^3^ Swiss Federal Institute for Forest, Snow and Landscape Research WSL, Birmensdorf, Switzerland; ^4^ Key Laboratory of Geographical Processes and Ecological Security in Changbai Mountains, Ministry of Education/School of Geographical Sciences, Northeast Normal University, Changchun, Jilin, China; ^5^ College of Life Science, Hebei University, Baoding, Hebei, China

**Keywords:** drought, clone plant, structural carbohydrate, non-structural carbohydrate, soil organic carbon fractions

## Abstract

**Introduction:**

The variety of organs carbon concentration may be important for tree survival rate, drought resistance and tree subsequent recovery. However, it remains unclear how drought affect structural carbohydrate (SC) and non–structural carbohydrate (NSC) export and transport on clonal plant, which can be correlated with sustain physiological metabolism and group drought resistance by resource sharing. To better understand the adaption ability of clone plants to drought and the linkage of organ carbon with soil organic carbon (SOC) fractions, we assessed how long–term drought affects organ carbon and its impact on SOC fractions among moso bamboo (*Phyllostachys edulis*) ramets.

**Methods:**

Throughfall exclusion included two treatments with simulated drought (TE) and control sample (CK, natural rainfall) by waterproofing panels, which excluded 70–80% of the precipitation. We measured the SC and NSC of leaves, branches and roots as well as soil organic carbon components on three ramets, which emerged in 2017 (grandma, GB), ramets that appeared in 2018 (mother bamboo, MB) and ramets emerging in 2019 (current-year bamboo, CB).

**Results:**

The results showed that there was significant difference on lignin, cellulose: lignin ratio and soluble sugar in leaves and roots (*p*<0.05) instead of branches (*p*>0.05). Effect of drought on SC and NSC varied with different organs and ramet age. Drought significantly increased soluble sugar concentration of leaves and roots by 15.5–31.0% and 10.6–24.8% for current-year bamboo. Compared with CK, drought decreased SOC by 6.7–19.1%, microbial biomass carbon (MBC) by 55.3–68.7%, readily oxidizable carbon (ROC) by 11.2–29.8%, particulate organic carbon (POC) by 25.1–47.4% but no effect on mineral–associated organic carbon (MOC). Drought changed the relationships of carbon components between plant organs and soil. In the control treatments, SC of leaves were significantly positively correlated with ROC, NSC of branches were positively correlated with ROC and MBC, NSC of roots were significantly positively correlated with SOC.

**Discussion:**

Overall, our results suggest that drought strengthened the linkage of plant organ carbon and soil carbon cycling among moso bamboo ramets in ecosystem studies, which are critical for predicting tree resistance and management in subtropical forest under drought.

## Introduction

Ongoing climate change as drought stress has exhibited a rising trajectory in recent years, leading to reduced ecosystem productivity and increased tree mortality (Salmon et al., 2019). Changes in drought patterns could potentially influence the capacity of the forest ecosystem in carbon sequester, potentially transitioning from carbon sink to carbon source, given the acknowledged role of drought in regulating soil carbon cycling (Chen et al., 2016). Drought inevitably influences the individual tree, and the carbon balance of ecosystems, intensity, and frequency of drought cause a strong decrease in the amount of newly absorbed carbon (Rog et al., 2024). Nonetheless, it has been confirmed that the variation in traits and response levels within and among species under drought conditions can mitigate the mortality rate and improve adaptation (O'Brien et al., 2014). However, the impacts of drought on soil organic carbon (SOC), particularly on its various components with plant-derived carbon quality, remain inadequately assessed within subtropical ecosystems.

The modification of plant structural carbohydrate (SC) caused by drought conditions is reported to play a significant role in enhancing plant resistance and defense (Hlahla et al., 2022). Generally, plants improve water uptake or reduce losses by resource-conservative shoot and root tissues with high structure characteristics (e.g., lignin content) with an avoidance drought strategy (Reinelt et al., 2023). Drought influenced the secondary wall by regulating the deposition of hemicellulose and lignin, with long-term drought generally leading to a profound impact on plant structure, resulting in thickening and lignification (Wang et al., 2023a). Drought has been found to decrease the synthesis of cellulose and its deposition in cotton (*Gossypium hirsutum*) (Ul-Allah et al., 2021), which results in thinning of cell walls and cell expansion due to plant osmotic pressure (Wang et al., 2016). Consequently, the change in SC is important for plant resistance to drought, particularly in signal transduction and cell wall architecture (Gall et al., 2015).

Non-structural carbohydrates (NSCs) primarily include starch and soluble sugars, which are important for transport, plant metabolism, osmotic adjustments, and serving as substrates for plant nutrient acquisition or defense (Hartmann and Trumbore, 2016). The concentrations and the relative contributions of organ-level NSC hold significance for plants’ annual phenological development (Montague et al., 2022), with NSC becoming increasingly critical under drought conditions (Kannenberg and Phillips, 2019). On the one hand, drought can lead to NSC depletion, causing an imbalance between carbon assimilation and respiration, ultimately resulting in tree mortality due to C starvation (O'Brien et al., 2014; Dulamsuren et al., 2023). On the other hand, the allocation amounts of NSC in organs, particularly roots, under drought conditions have been linked to tree survival, resistance, and forest resilience by osmoregulation and hydraulic function (Canham et al., 1999; O'Brien et al., 2014). The drought adaptation mechanism of NSC and the conversion relationships from sugars to starch in clonal plant ramets remain unclear. Therefore, understanding the impact of drought on the NSC dynamic is crucial for unraveling the mechanisms of drought recovery and the fundamental pattern of new carbon allocation in trees (Wang et al., 2021a; Kannenberg and Phillips, 2019).

Severe drought has a significant impact on the storage, stability, and processes of terrestrial carbon (Deng et al., 2021). Drought not only impacts aboveground C processes (Gao et al., 2021) but also influences belowground SOC fraction and stability (Sun et al., 2023; Novair et al., 2024). It has been observed that drought diminishes the quantity and quality of the SOC (Su et al., 2020), leading to a 3.3% reduction in SOC and a 59% increase in dissolved organic carbon as a meta-analysis (Deng et al., 2021). Long-term drought experiments have shown a significant reduction in soil particulate organic carbon (POC) and an increase in mineral-associated organic carbon (MOC) in the 0–10-cm layer for plant-derived carbon (Sun et al., 2023). Additionally, variation in the quality of dissolved organic carbon under drought can further regulate soil microbial metabolism and nutrient transformation (Tiwari et al., 2022). Examining the effects of drought on the quantity and quality of SOC is essential for understanding the dynamics of forest SOC pools under long-term drought (Su et al., 2020). However, drought-induced changes in the carbon linkage between above- and below-ground processes depend on SOC stability, leading to unpredictable outcomes (Wang et al., 2021b). Therefore, quantifying a threshold for improvement and formulating strategies to alleviate drought’s impact on forest growth is important for sustainable ecological provision in subtropical forest management.

Moso bamboo (*Phyllostachys edulis*) is extensively distributed in subtropical regions of China, covering approximately 4.68 million hectares, and it exhibits significant potential for carbon sequestration and economic advantages (Ge et al., 2022; Ouyang et al., 2022). The increasing frequency of drought occurrences in moso bamboo habitats in recent years is likely to impact the cultivation and management level of moso bamboo (Tong et al., 2020). Compared to the pot experiment, the benefits of plant–soil carbon association through whip root system exploitation and deeper soil water acquisition under drought conditions will be more credible and authentic for clonal plants in the field with comprehensive studies. Generally, mature trees with larger rooting systems have access to deeper water stores, which leads to less impact on their photosynthetic efficiency and resource acquisition as compared to younger trees (Arain et al., 2022). The impact of drought-induced alterations in organ carbon on SOC fractions within different-aged moso bamboo ramets remains uncertain. Therefore, we hypothesize that 1) drought induces significant differences in SC and NSC among ramets and 2) the changes in SC and NSC in organs would lead to fluctuations in SOC fractions and stability to adapt to drought conditions. The study would provide the plant–soil carbon feedback on drought to improve the measures of combatting drought and future research directions.

## Materials and methods

### Study site

**Figure 1:**
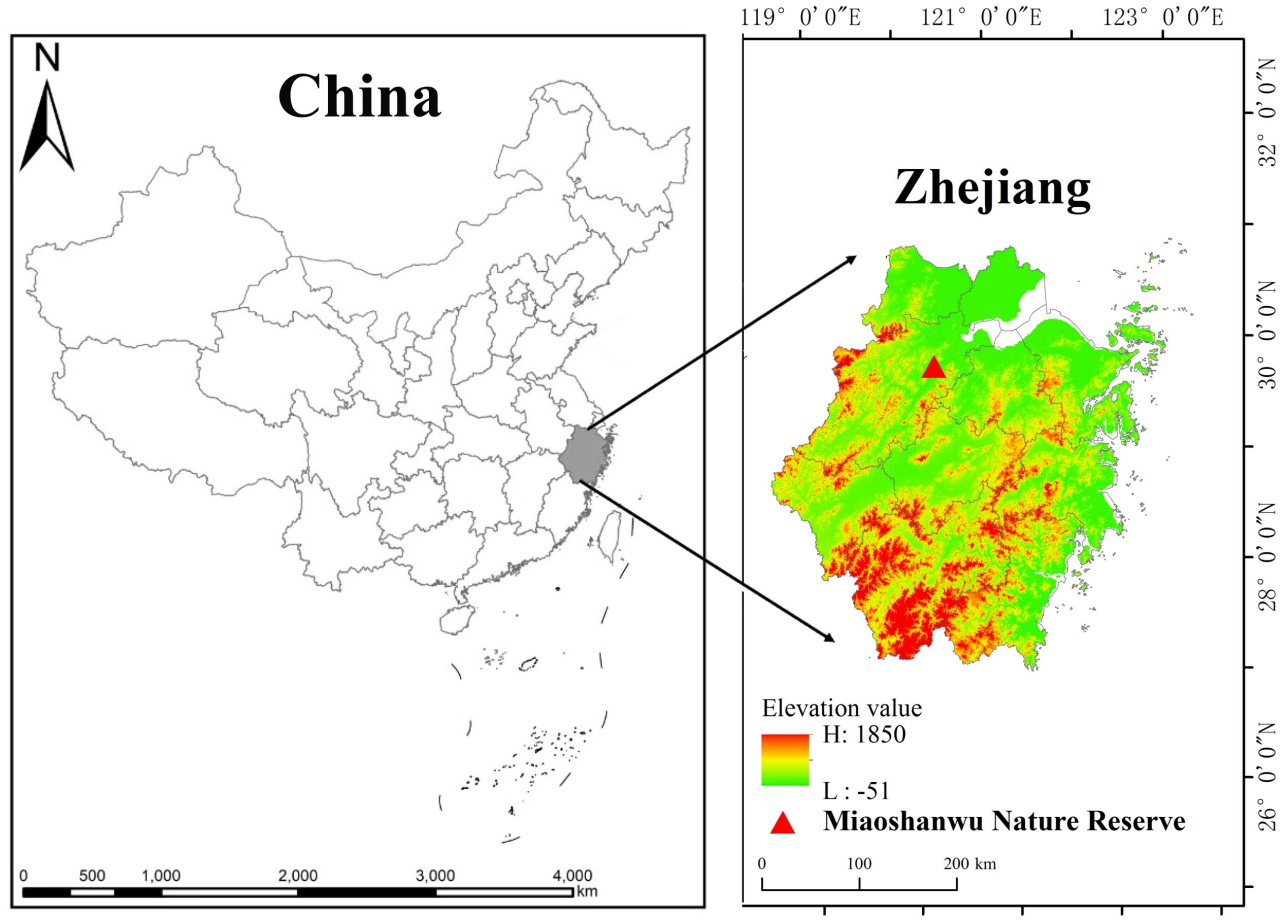
The study area locates in the Miaoshanwu nature reserve, Zhejiang Province, China.

This study was conducted at the Qianjiangyuan Forest Ecosystem Research Station of the State Forestry and Grassland Administration of China (30°06′ N, 120°00′ E), situated in Fuyang District, Hangzhou, China (**Figure 1**). The forest area of Miaoshanwu spans approximately 536.7 hm^2^, with a core area of 348.8 hm^2^, and encompasses various vegetation types including moso bamboo plantations, *Cunninghamia lanceolata*, *Pinus massoniana*, natural secondary forests, and shrub forests, with moso bamboo plantations occupying 30% of the total area. The altitude ranges from 11 m to 536 m, and the soil is classified as reddish loam with a pH of 5.72. The study area exhibits a typical subtropical monsoon climate, with an annual mean temperature of 16.9°C, a mean temperature of 25.4°C in the hottest month, and a mean temperature of 4.5°C in the coldest month. The mean annual precipitation is 1,513 mm, the annual duration of sunshine is 1,995 h, and the annual frost-free period lasts for 237 days.

### Experimental design and sampling

Three moso bamboo plantations with similar site conditions were identified within the Miaoshanwu forest station. A single fixed plot measuring 10 m × 25 m was selected in each moso bamboo plantation in 2014, subsequently partitioned into two subplots measuring 10 m × 10 m, designated for throughfall exclusion (TE; simulated drought) and control sample (CK; natural rainfall). To mitigate any potential interference, a 5-m spacing was maintained between the two subplots, and the TE and CK treatments were replicated three times. Following the end of the bamboo shoot period in July 2014, PVC waterproofing panels were erected as roofs over drought treatments at a height of 1.5 m from the ground (with an 11 m × 11 m area for the marginal effect), which excluded 70%–80% of the precipitation. Trenches (50 cm long, 20 cm wide, and 60 cm deep) were excavated around the drought subplots to minimize boundary effect and improve drainage, with the interior of the trenches being lined with PVC plastic. The control subplots were subjected to excavation of trenches of identical dimensions, devoid of any additional treatments. The monthly dynamic data of rainfall, soil temperature, and moisture in 0–60 cm (divided by 0–10 cm, 10–20 cm, 20–40 cm, and 40–60 cm) were measured using EM50 (Decagon, San Francisco, CA, USA) in each subplot (**Supplementary Figure S1**). The characteristics of the study plots are presented in **Table 1**.

**Table 1:** General information for the control plots (CK) and the throughfall exclusion plots (TE) in the experimental moso bamboo stands (mean ± SD, *n*=3).

Factors	Stand 1		Stand 2		Stand 3	
	CK	TE	CK	TE	CK	TE
Elevation (m a.s.l.)	136	136	141	141	146	146
Slope (°)	15	15	20	20	22	22
Slope aspect	S	S	S	S	S	S
Soil depth (cm)	60	60	60	60	60	60
SOC (g·kg^−1^)	34.37 ± 9.06	36.11 ± 7.03	36.74 ± 8.04	39.78 ± 7.39	37.82 ± 8.21	37.42 ± 9.64
Soil total N (g·kg^−1^)	2.72 ± 0.79	2.34 ± 0.27	2.72 ± 0.48	2.91 ± 0.52	2.56 ± 0.29	2.52 ± 0.41
SAP (mg·kg^−1^)	4.25 ± 0.93	4.64 ± 0.81	5.50 ± 1.03	5.94 ± 1.23	6.48 ± 1.09	6.45 ± 2.04
DBH (cm)	13.42 ± 2.17	14.72 ± 1.55	14.44 ± 2.14	14.30 ± 1.93	12.99 ± 2.53	14.60 ± 1.26
Height (m)	17.28 ± 0.90	16.32 ± 2.28	16.97 ± 1.50	15.97 ± 2.04	15.80 ± 2.41	16.64 ± 0.86
Density (n·ha^−1^)	3,800	3,600	3,400	3,000	3,250	2,800

S, sunny slope; SOC, soil organic carbon; SAP, soil available phosphorus; DBH, diameter at breast height.

Bamboo ramets interconnected through rhizomes were categorized into groups based on the year of sprouting: ramets that emerged in 2017 (grandma; GB), ramets that appeared in 2018 (mother bamboo; MB), and ramets emerging in 2019 (current-year bamboo; CB). In August 2019, fresh leaves, branches, and roots were gathered from both the TE and CK treatment subplots. Leaves and branches were collected from four orientations, while roots were manually excavated and rinsed with deionized water. Each series of samples obtained from the identical subplot was aggregated to create a composite sample with five replicates. The freshly collected samples were first subjected to a 30-min drying period at 105°C, followed by further drying at 70°C for 48 h until a consistent weight was achieved.

Concurrently, six rhizosphere soil samples were collected from each subplot at depths of 0–20 cm through manual excavation, with five repeated measurements. All fresh soil samples were sieved through a 2-mm mesh after the removal of fine roots and visible debris. Subsequently, the composite samples underwent natural air-drying and were subsequently shifted to a 0.25-mm mesh in preparation for SOC fraction analysis.

### Sample analysis

The quantification of lignin and cellulose concentration was measured by acid washing (Wang et al., 2016). The determination of acid-soluble lignin content was measured at 205 nm using a UV spectrophotometer (UV-1800, ESM, China) and a muffle oven (CR7, Nanjing, China). In parallel, the insoluble lignin content was ascertained by subjecting the solid material to drying until a constant mass at 105°C ± 3°C using a muffle furnace (CR7, Nanjing, China). Subsequently, the relative lignin content was estimated according to the above two values, and the test was replicated twice for each sample.

NSC in moso bamboo encompasses soluble sugars and starch content, as per the definition adopted in this study. The NSC analysis followed the approach outlined by Li et al. (2008), with each sample being subjected to duplicate analyses and the mean values used to represent the NSC content of the sample. Several adjustments were implemented to ascertain the NSC content. Specifically, 0.10-g samples from various organs were accurately weighed and transferred into 10-mL centrifuge tubes. Subsequently, 2 mL of an 80% ethanol solution was introduced to the centrifuge tube and subsequently subjected to a 30-min water bath at 80°C. Upon cooling to ambient temperature, the solution was centrifuged at a speed of 4,800 r·min^−1^ for a duration of 10 min, the supernatant was retained to determine the soluble sugar content, while the precipitate was preserved for determination of starch content. This extraction process was repeated three times. The precipitate was then subjected to the addition of 2 mL of distilled water, followed by gelatinization in a boiling water bath for 15 min. Upon reaching room temperature, 2 mL of 9.2 M HClO_4_ solution was added, followed by shaking for 15 min and subsequent addition of 4 mL of distilled water. The resulting mixture was then centrifuged at 4,800 r·min^−1^ for 10 min. After aspiration of the supernatant, a further extraction with 2 mL of 4.6 M HClO_4_ was performed. The entirety of the supernatants was gathered for the purpose of ascertaining the starch content. The absorbance of the solution resulting from the reaction between sugar and starch with the anthrone reagent was quantified using a spectrophotometer set at 625 nm. The content was quantified using the standard curve and expressed as a percentage relative to the organs’ dry weight.

SOC was measured by an elemental analyzer (Elementar, Langenselbold, Germany). The determination of POC was performed according to the method of Cambardella and Elliott (1992). Specifically, 15 g of 0.25-mm air-dried soil was mixed with 100 mL of a 5 g·L^−1^ sodium hexametaphosphate solution. This mixture was then subjected to agitation on a reciprocal shaker with 90 r·min^−1^ for 18 h after manually shaking the above mixture for 5 min. Subsequently, the uniformly mixed soil sample was sieved through a 53-μm soil sieve and thoroughly washed with distilled water. The material adhering to the 53-μm soil sieve was designated as the POC fraction, while the soil sample passing through the 53-μm soil sieve was regarded as the MOC fraction. Subsequently, both soil samples were dried at 60°C and then prepared for the determination of organic carbon using an organic carbon analyzer (Shimadzu Corp., Kyoto, Japan). The determination of readily oxidizable carbon (ROC) was conducted using 333 mol·L^−1^ KMnO_4_ oxidation colorimetry (Blair et al., 2019). The measure of soil microbial biomass carbon (MBC) was carried out following the chloroform fumigation–extraction method (Vance et al., 1987).

### Statistical analysis

Data analysis was conducted using the SigmaPlot 11.0 and SPSS 16.0 software. The t-test was employed to compare organ carbon and SOC fractions between control and drought conditions. After a normality test and Mauchly’s test, the ramet age variations of the parameters in relation to the treatment were analyzed using treatment and ramet age as fixed factors. For the comparison of means, one-way analysis of variance (ANOVA) and least significant difference (LSD) were utilized for multiple comparisons on NSC, SC, and SOC fractions in the different ramets at a significance level of *α* = 0.05. A two-way ANOVA was employed to assess the impact of treatments and ramet age on organ carbon as well as SOC fractions. Correlation analysis and the Mantel test were utilized to ascertain the relationships between organ carbon and SOC fraction. Graphs were generated using the SigmaPlot 11.0 software, with data presented as mean ± standard deviation.

## Results

### Effect of long-term drought on structural carbohydrate in different organs

**Figure 2:**
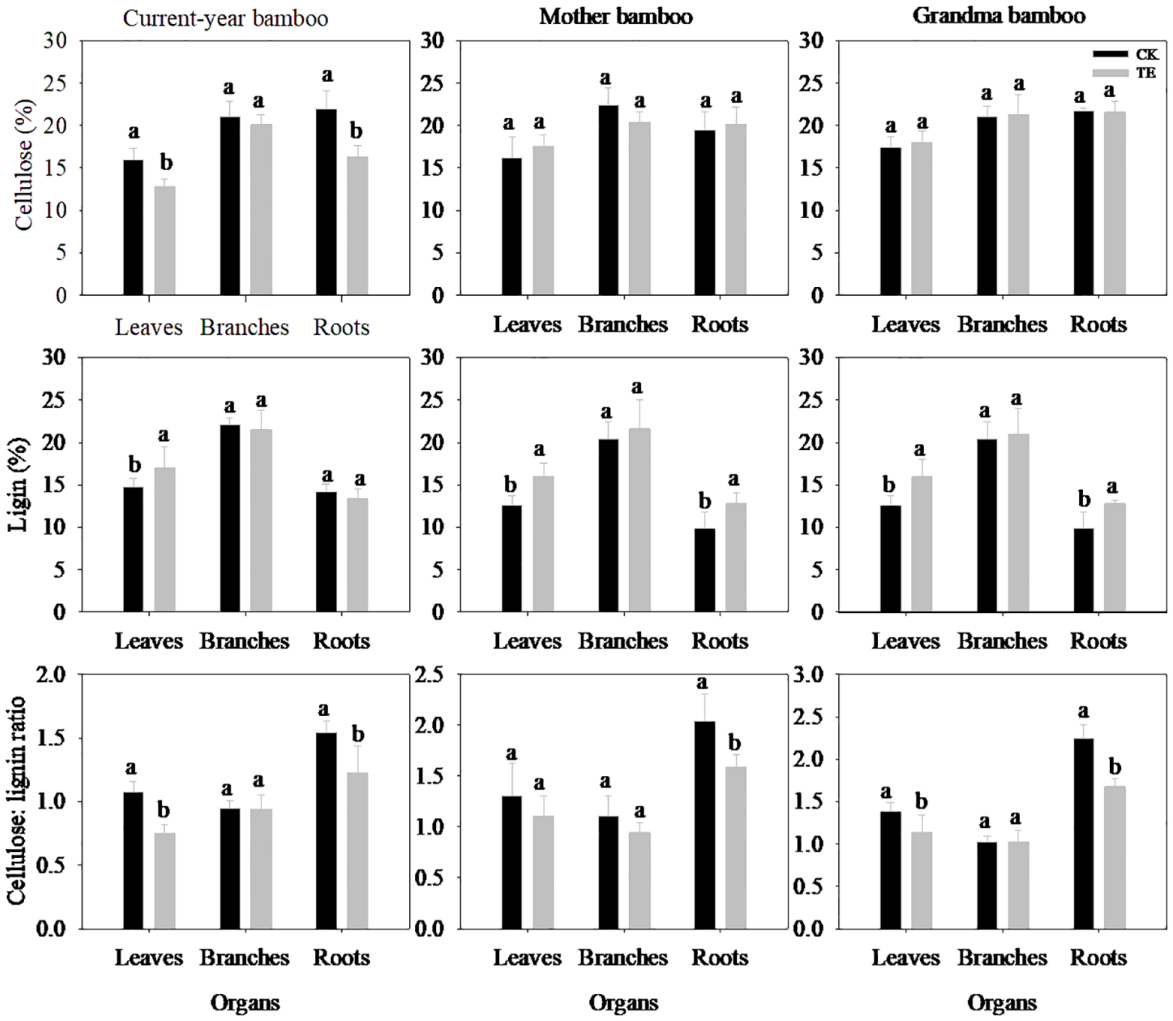
Effects of rainfall manipulation treatment (TE vs. CK) on cellulose, lignin and the ratio of cellulose: lignin in different organs of moso bamboo plantations. Different letters in the same organ mean significant difference at 0.05 level between CK and TE treatments.

The impact of drought on cellulose and lignin exhibited organ-specific variations (**Figure 2**; **Table 2**). Under drought conditions, no significant effect on cellulose concentration was observed across all organs (**Table 2**, *p* > 0.05). However, a significant difference was noted in the lignin and cellulose:lignin ratio in leaves and roots (**Table 2**, *p* < 0.05), whereas no such difference was evident in branches (*p* > 0.05). Specifically, drought led to a substantial reduction in cellulose concentration in leaves and roots by 20.0% and 25.5% for current-year bamboo, respectively. Conversely, it resulted in an increase in the lignin concentration of three ramets in leaves by 15.2%, 26.9%, and 26.9% and in roots by 29.7% for mother and grandma bamboo (no effect on current-year bamboo).

**Table 2:** Effects of rainfall manipulation treatment (*TE* vs. *CK*), bamboo age (*A*) and their interactive effects on cellulose, lignin, and the ratio of cellulose:lignin in different organs of moso bamboo plantations across the sampling period, analyzed with two-way analysis of variance.

Effects	Rainfall treatment (*R*)			Age (*A*)			*R* × *A*		
	*d*_f_	*F*	*p*	*d*_f_	*F*	*p*	*d*_f_	*F*	*p*
Cellulose										
Leaves	1	0.384	0.547	1	8.044	**0.006**	1	3.853	0.051
Branches	1	1.300	0.276	1	0.353	0.710	1	0.701	0.515
Roots	1	4.637	0.052	1	3.595	0.060	1	6.324	**0.013**
Lignin										
Leaves	1	14.824	**0.002**	1	1.886	0.194	1	0.236	0.793
Branches	1	0.118	0.737	1	0.323	0.730	1	0.223	0.803
Roots	1	7.221	**0.020**	1	6.982	**0.010**	1	4.024	**0.046**
Cellulose:Lignin ratio									
Leaves	1	8.890	**0.011**	1	6.247	**0.014**	1	0.186	0.833
Branches	1	1.148	0.305	1	0.956	0.412	1	0.804	0.470
Roots	1	33.611	**0.000**	1	20.296	**0.000**	1	0.902	0.432

*F* and *p* values are given. The bold value meant significant difference at 0.05 level.

The response of cellulose and lignin concentrations to drought varied among different ramets (**Figure 2**; **Table 2**). Notably, significant differences in both cellulose and lignin concentrations were observed in leaves (*p* < 0.05) of three ramets, as opposed to branches (*p* > 0.05) and roots (*p* > 0.05). With regard to the cellulose:lignin ratio, there were significant differences in leaves (*p* = 0.014) and roots (*p* = 0.000), but not in branches (*p* > 0.05). Moreover, the interaction effect between treatment and ramet age on cellulose and lignin concentrations in roots was evident across the study period.

### Effect of long-term drought on non-structural carbohydrates in different organs

**Figure 3:**
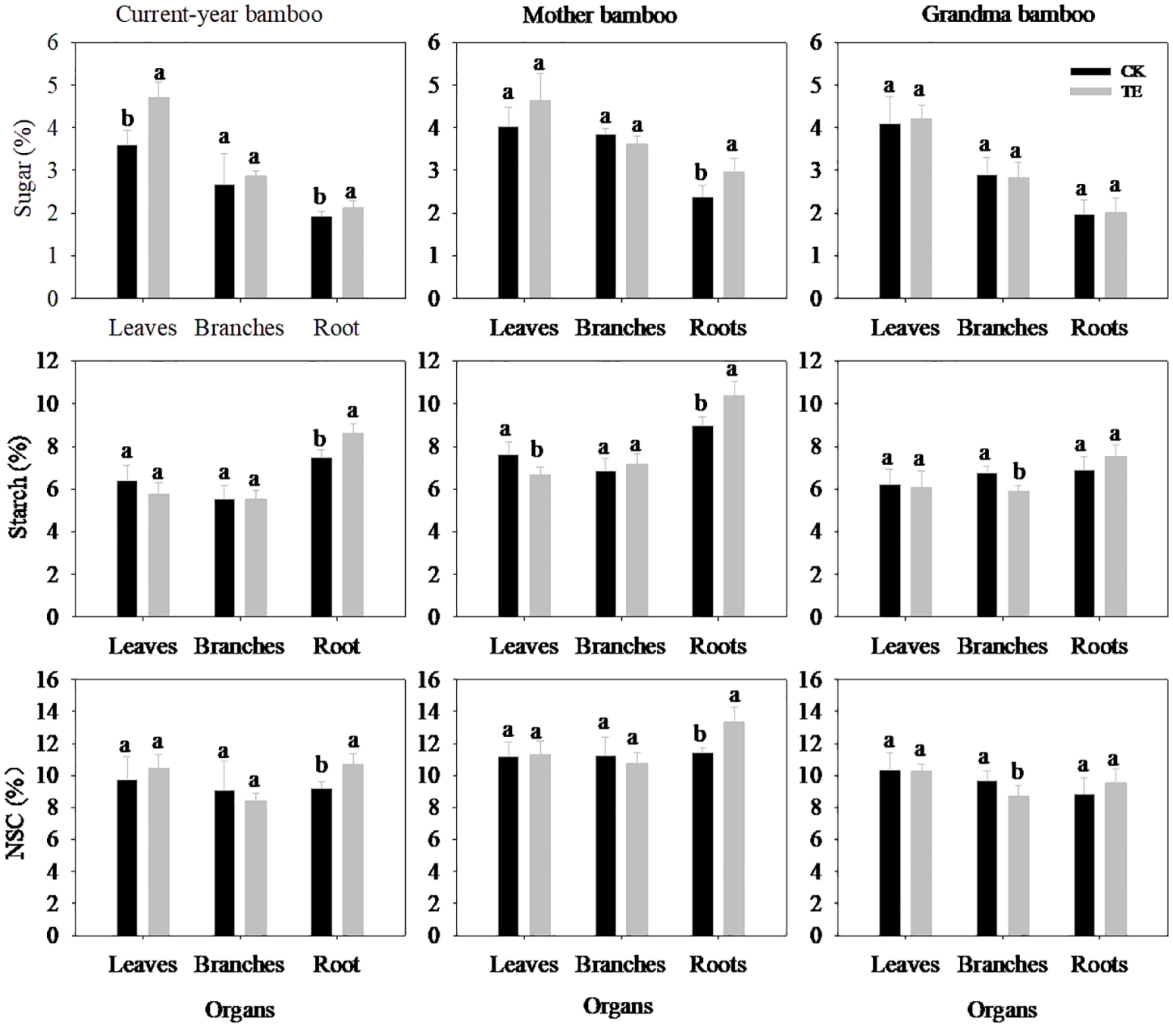
Effects of rainfall manipulation treatment (TE vs. CK) on sugar, starch and non-structural carbohydrate in different organs of moso bamboo plantations. Different letters in the same organ mean significant difference at 0.05 level between CK and TE treatments.

The impact of drought on NSC levels varied across different organs and ramet ages (**Figure 3**; **Table 3**). Notably, the concentration of soluble sugar in leaves and roots was significantly affected under drought conditions (**Table 3**, *p* < 0.05), while no significant impact was observed in branches (*p* > 0.05). Drought led to a substantial increase in the concentration of soluble sugar concentration of leaves and roots by 31.02% and 10.59% for current-year bamboo and by 15.47% and 24.76% for mother bamboo, respectively. Furthermore, drought had a significant effect on starch and NSC concentrations only in roots with increases of 15.21% and 15.48% for current-year bamboo, 9.24% and 16.50% for mother bamboo, 16.30% and 7.68% for grandma bamboo, respectively.

**Table 3:** Effects of rainfall manipulation treatment (*TE vs. CK*), bamboo age (*A*) and their interactive effects on soluble sugar, starch, and non-structural carbohydrate in different organs of moso bamboo plantations across the sampling period, analyzed with two-way analysis of variance.

Effects	Rainfall treatment (*R*)			Age (*A*)			*R* × *A*		
	*d*_f_	*F*	*p*	*d*_f_	*F*	*p*	*d*_f_	*F*	*p*
Soluble sugar									
Leaves	1	7.676	**0.017**	1	0.300	0.746	1	1.723	0.220
Branches	1	0.025	0.876	1	11.244	**0.002**	1	0.466	0.639
Root	1	0.4705	**0.049**	1	12.147	**0.001**	1	1.593	0.243
Starch									
Leaves	1	4.009	0.068	1	5.646	**0.019**	1	0.609	0.560
Branches	1	0.685	0.424	1	15.973	**0.000**	1	2.824	0.099
Root	1	19.355	**0.001**	1	35.976	**0.000**	1	0.847	0.453
Non-structural carbohydrate									
Leaves	1	0.326	0.578	1	2.562	0.118	1	0.287	0.756
Branches	1	2.302	0.155	1	9.016	**0.004**	1	0.085	0.919
Root	1	15.895	**0.002**	1	31.921	**0.000**	1	1.071	0.373

*F* and *p* values are given. The bold value meant significant difference at 0.05 level.

Significant differences were observed in the concentrations of soluble sugar, starch, and NSC concentrations among different ramets (**Figure 3**; **Table 3**). Specifically, starch concentration was significantly different in leaves (*p* = 0.019), branches (*p* = 0.000), and roots (*p* = 0.019). Moreover, for soluble sugar and NSC, there were significant differences in branches (*p* = 0.002 and *p* = 0.004) and roots (*p* = 0.001 and *p* = 0.000). However, no interaction effect between treatment and ramet age on the concentrations of soluble sugar, starch, and NSC in all organs was observed throughout the study period.

### Effect of long-term drought on soil organic carbon components

**Figure 4:**
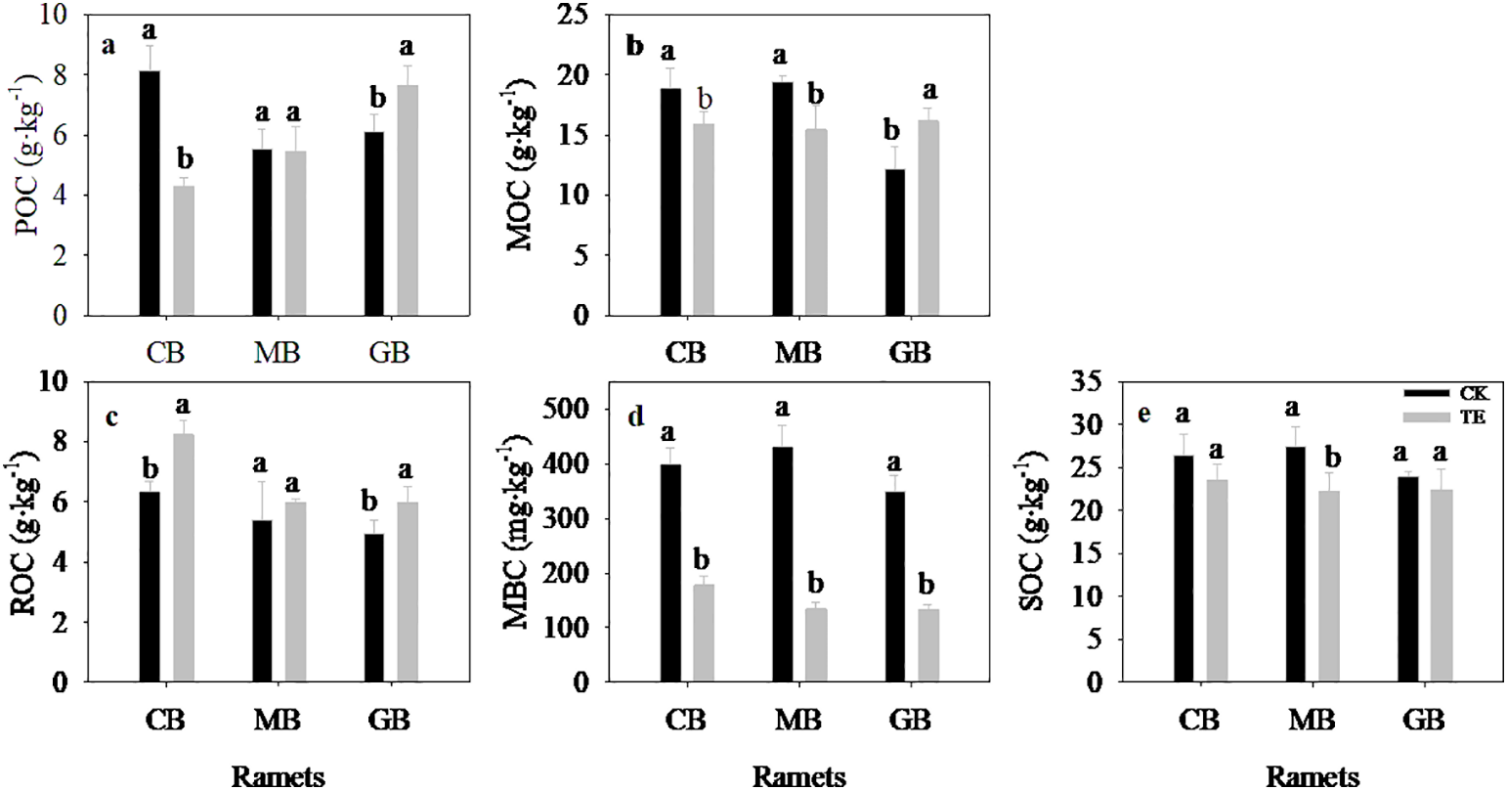
Effects of rainfall manipulation treatment (TE vs. CK) on soil organic carbon fractions in moso bamboo plantations. POC, soil particulate organic carbon; ROC, readily oxidizable carbon; MBC, microbial biomass carbon; MOC, mineral-associated organic carbon. Different letters in the same ramet mean significant difference at 0.05 level between CK and TE treatments.

Drought had a significant impact on SOC fractions except for MOC (**Figure 4**; **Table 4**, *p* = 0.005). In comparison to control treatments, the level of SOC decreased by 10.9%, 19.1%, and 6.7% for current-year bamboo, mother bamboo, and grandma bamboo, while MBC decreased by 55.3%, 68.7%, and 61.6%, respectively. Additionally, drought led to an increase in ROC by 29.8%, 11.2%, and 21.2% for current-year bamboo, mother bamboo, and grandma bamboo, respectively. Pertaining to POC, drought caused a decrease of 47.4% for current-year bamboo and an increase of 25.1% for grandma bamboo. However, for MOC, drought resulted in a decrease of 15.7% and 20.5% for current-year bamboo and mother bamboo, respectively, and an increase of 32.2% for grandma bamboo.

**Table 4:** Effects of rainfall manipulation treatment (*TE vs. CK*), bamboo age (*A*) and their interactive effects on soil particulate organic carbon and mineral organic carbon in rhizosphere soil of moso bamboo plantations across the sampling period, analyzed with two-way analysis of variance.

Effects	Rainfall treatment			Ramet ages			Treatments×ramet ages		
	*d*_f_	*F*	*p*	*d*_f_	*F*	*p*	*d*_f_	*F*	*p*
Particulate organic carbon	1	6.660	**0.024**	2	6.374	**0.013**	2	25.896	**0.000**
Mineral organic carbon	1	2.211	0.163	2	10.147	**0.003**	2	13.632	**0.001**
Readily oxidizable carbon	1	15.124	**0.002**	2	14.407	**0.001**	2	1.552	0.251
Microbial biomass carbon	1	445.246	**0.000**	2	6.227	**0.014**	2	5.059	**0.026**
Soil organic carbon	1	11.541	**0.005**	2	1.578	0.246	2	1.276	0.314

*F* and *p* values are given. The bold value meant significant difference at 0.05 level.

The ages of ramet exhibited a significant impact on SOC fraction except SOC (**Figure 4**; **Table 4**, *p* = 0.005). Under control treatments, soil carbon fractions (excluding MBC) in current-year bamboo were significantly higher than those in grandma bamboo. In drought conditions, ROC and MBC in current-year bamboo were significantly higher than those in mother bamboo (by 27.3% and 24.2%, respectively) and grandma bamboo (by 27.4% and 24.4%, respectively) (**Table 4**). Moreover, POC in current-year bamboo was significantly lower than in mother bamboo (26.9%) and grandma bamboo (77.7%). There was a significant interaction effect between drought and ramet ages for POC, MOC, and MBC (*p* < 0.05) (**Table 4**, *p* < 0.05).

### Drought on the relationships between organ carbon and SOC components

**Figure 5:**
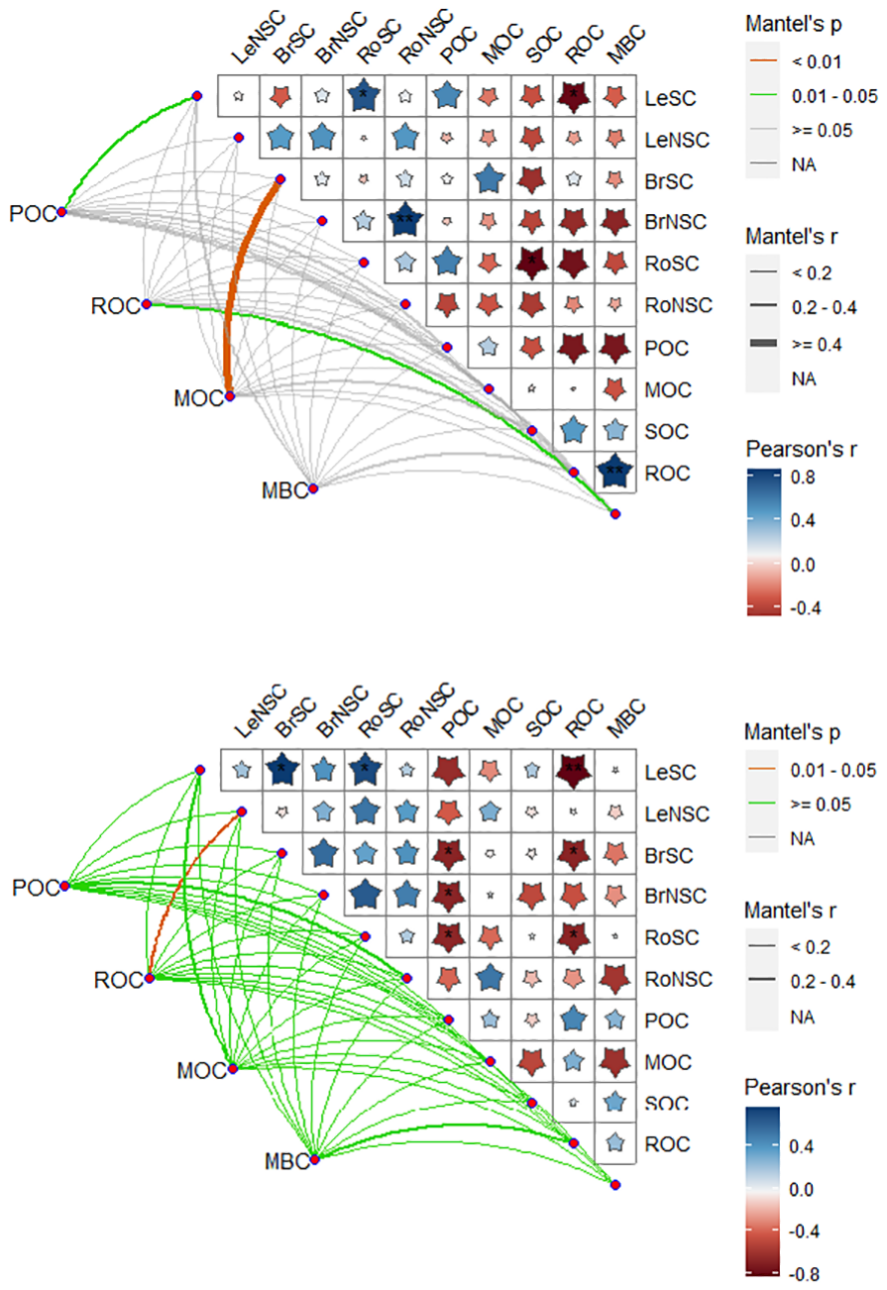
Correlation between organ carbon and soil organic carbon fractions under rainfall manipulation treatment and control (TE vs. CK) in moso bamboo plantations. Blue and red in the square represent positive and negative correlations between the two variables, respectively. The deeper the color, the stronger the relationship. POC, soil particulate organic carbon; ROC, readily oxidizable carbon; MBC, microbial biomass carbon; MOC, mineral-associated organic carbon; LeNSC, non-structural carbon in leaves; BrSC, structural carbon in branches; BrNSC, non-structural carbon in branches; RoSC, structural carbon in roots; RoNSC, non-structural carbon in roots. *p < 0.05; **p < 0.01.

Drought changed the relationships of carbon components in plant organs and soil (**Figure 5**). Under the control treatments, the SC of leaves showed a significant positive correlation with ROC, while the NSC of branches exhibited a positive correlation with ROC and MBC. Furthermore, the NSC of roots was significantly positively correlated with SOC. Additionally, MOC displayed a positive correlation with ROC and MBC, while ROC exhibited a negative correlation with MBC.

**Figure 6:**
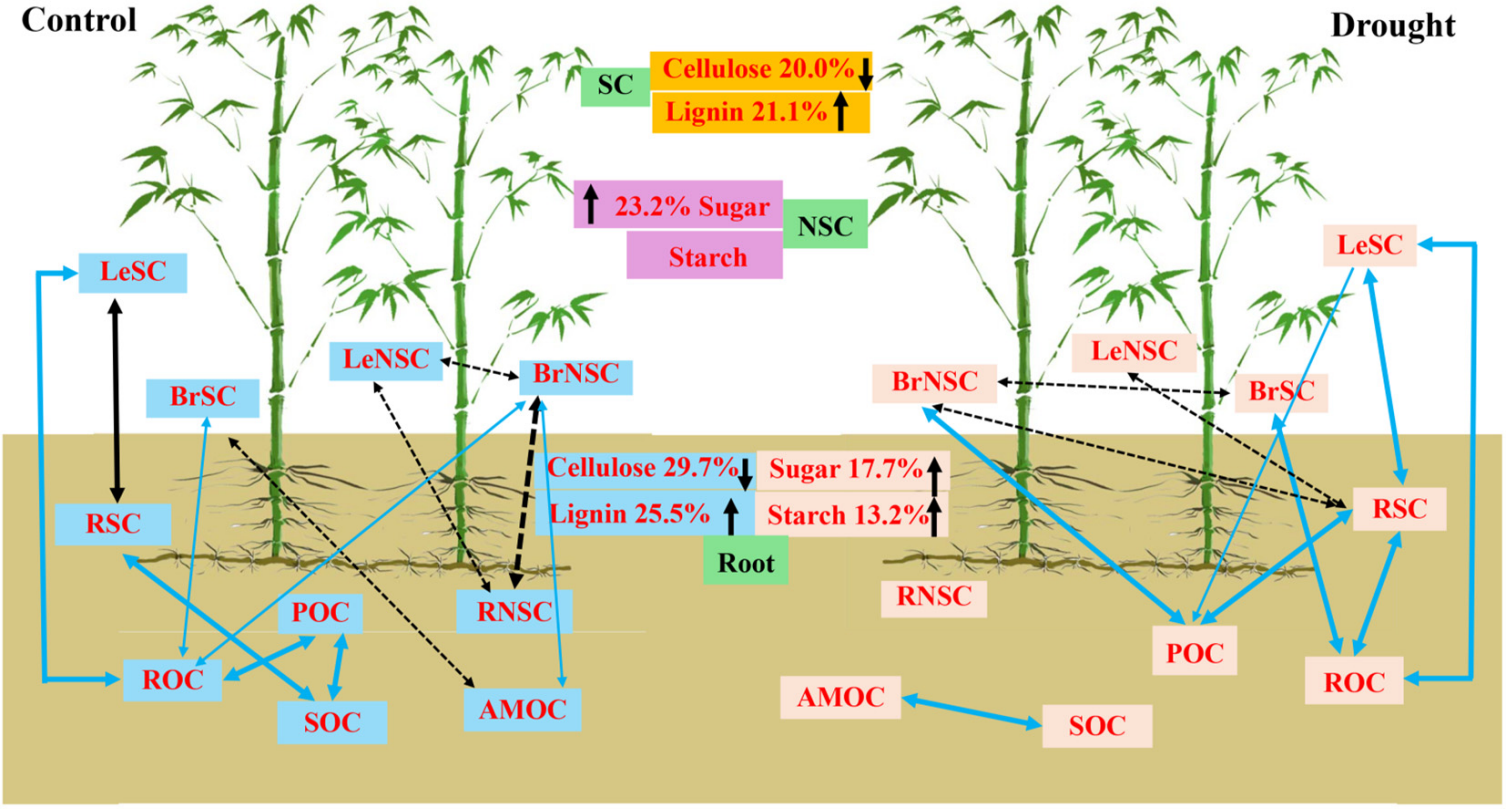
Schematic representation of the linkage of organ carbon and soil organic carbon fractions under rainfall manipulation treatment (TE vs. CK) on moso bamboo plantations. Blue and black in the square represent positive and negative correlations between organ carbon and SOC fractions variables, respectively. The solid line represents significance, dashed line represents insignificance. The black upward arrow indicates an increase, the black downward arrow indicates a decrease. POC, soil particulate organic carbon; ROC, readily oxidizable carbon; MBC, microbial biomass carbon; MOC, mineral-associated organic carbon; LeNSC, non-structural carbon in leaves; BrSC, structural carbon in branches; BrNSC, non-structural carbon in branches; RoSC, structural carbon in roots; RoNSC, non-structural carbon in roots.

The drought conditions strengthened the connections between SC and NSC in various tissues and organs of moso bamboo with SOC fractions (**Figure 5**). Under the drought treatments, the SC of leaves exhibited a significant positive correlation with ROC, while the SC of branches showed a positive correlation with ROC and POC. Additionally, the NSC of branches displayed a positive correlation with POC, and the SC of roots was associated with POC and ROC. Moreover, the NSC of roots showed a significant positive correlation with MBC. Furthermore, the SC of leaves was significantly correlated with the SC carbohydrates of branches and roots. POC was positively linked with MBC (**Figure 6**).

## Discussion

### Effect of drought on SC in different ramets

The impact of drought stress on cell wall composition remains relatively limited, despite its evident effects on cell wall (van der Weijde et al., 2017). Drought caused the shift of fiber fractions (cellulose and lignin) with more resource-conservative morphological traits, instead of becoming tougher (Reinelt et al., 2023). In *Miscanthus sinensis*, a reduction in cell wall content in leaves of 6%–8% under 3 weeks of drought was observed, particularly affecting SC such as cellulose and hemicellulose (Hassan et al., 2022). In a recent study, the subsequent increase in cellulose proportion by 2.2% and 3.2% after 3 weeks of drought did not affect leaf lignin levels, suggesting a downregulation of cellulose biosynthesis in response to drought (Hassan et al., 2022). Moreover, an increase in cellulose content in the branch indicated enhanced secondary growth due to drought (Rehschuh et al., 2022). However, in the current study on moso bamboo, no significant effect on cellulose in all organs under drought conditions (**Table 2**, *p* > 0.05) was observed, suggesting that drought has a limited affected on the thickness and hardness of the cell wall in moso bamboo organs. In contrast, van der Weijde et al. (2017) demonstrated a substantial decrease in cellulose content in stem tissue by 46%–51% and leaf tissue by 43% in *Miscanthus* under drought conditions. Furthermore, the study by Hlahla et al. (2022) revealed that drought significantly decreased cellulose at 3,800–3,000 cm^−1^ and lignin at 1,458 cm^−1^. These inconsistent results indicate that clonal plant has different drought adaptation strategies by resource sharing of whip root system without limiting chemical trait response (Tong et al., 2020).

The lignin content in roots is related to root mechanical flexibility, water uptake, and soil carbon sequestration by roots (Suseela et al., 2020). Drought has been found to significantly increase soluble lignin content by 17%–27% (Hlahla et al., 2022). In this study, significant differences in lignin and cellulose:lignin ratio were observed in leaves and roots (**Table 2**, *p* < 0.05), but not in branches (*p* > 0.05), indicating that the changes in chemical structure composition in leaves and roots are an important resource-conservative strategy of drought resistance (Suseela et al., 2020; Eberhardt and Samuelson, 2022). Both increases and decreases in lignin contents under drought conditions have been reported in different tissues and plant species. van der Weijde et al. (2017) found that drought had significantly affected stem lignin content (*p* = 0.015), but not leaf tissues (*p* = 0.522) in *Miscanthus*. The inconsistent findings suggested that the levels of lignin may either improve or reduce depending on the plant and tissue type. The change in structural rigidity facilitates the improvement of vertical growth and resistance to pathogenic and excessive transpiration (Wang et al., 2023a). Changes in SC concentration in moso bamboo indicated that enhancing lignin deposition, which enhances physical resistance to drought, could be utilized as an alternative approach to cultivating drought-resistant plants (Choi et al., 2023). Furthermore, in this study, compared with old ramets, young ramet had higher lignin and lower cellulose:lignin ratio in all organs, suggesting that young bamboo possesses stronger mechanical strength and stability of the cell wall to protect cells from external damage (Shi et al., 2022).

### Effect of drought on NSC in different ramets

Drought led to a great decrease in leaf starch concentration and cut down the transport of new carbohydrates from aboveground to belowground (Huang et al., 2024). The responses of NSC concentration to drought in trees vary with drought intensity and duration as well as tree species (Thompson et al., 2023). In the present study, drought significantly increased the soluble sugar concentration of leaves (15.5%–31.0%), suggesting that drought caused an increase in carbon transportation to belowground tissues by the level of root carbon demand for fine root expansion (Liu et al., 2020); this indicates a conversion between leaf starch and soluble sugars for meeting the demand of osmotic regulation with a decrease in leaf water potential under drought (Huang et al., 2024). Similarly, the study by Wang et al. (2021c) showed that drought increased the soluble sugars content and enhanced the soluble sugars:starch ratio in all tissues of *Pinus tabulaeformis* seedlings but decreased the starch concentration with the increase in the drought intensity. In this study, the change in NSC components in roots confirmed that drought increased the downward translocation of aboveground organs for photosynthetic production (Wang et al., 2021a; Kono et al., 2019), suggesting that lower levels of soluble sugars in leaves may retain new photosynthates for fast turnover to meet the demand of basal respiration or other production (e.g., monoterpenes) (Alagoz et al., 2023; Huang et al., 2024). In contrast, a meta-analysis of 71 tree species showed that drought significantly reduced NSC in roots by 17.3% (Li et al., 2018). Therefore, the change in NSC under drought conditions depends on tree type, tree age, and leaf habit (Li et al., 2018).

In this study, drought led to a significant increase in soluble sugar (10.6%–24.8%), starch (9.2%–15.5%), and NSC (7.7%–16.5%) levels in roots (**Figure 3**). The rise in NSC levels in roots due to drought may suggest that improved carbon translocation increased more assimilated carbon toward root utilization for metabolic requirements (Li et al., 2018). This result indicates that roots play an important role in maintaining survival and improving drought resistance by regulating the NSC of the whip root system for clonal plants (Stefaniak et al., 2024; Xiao et al., 2024). It appears that the reduction in NSC availability to tree roots occurs at a faster rate than NSC utilization during water stress conditions. Conversely, Kannenberg and Phillips (2019) showed that NSC storage remained remarkably stable across different species and tissues under drought conditions despite a drastic decrease in carbon assimilation. Consequently, roots may encounter carbon deficiency or adjust the NSC storage prior to other plant parts (Stefaniak et al., 2024), offering insights for enhancing mechanistic forecasts of tree mortality in Dynamic Global Vegetation Models (DGVMs) (Deng et al., 2021).

In this study, the age of ramets was found to significantly impact soluble sugar and NSC levels in both branches and roots as well as starch in all organs (*p* < 0.05), indicating that old NSC storage reserves in stem and roots have more important role in sustaining survival and metabolism under drought conditions (Huang et al., 2024). In this study, the lower NSC levels in young bamboo for all organs suggest that the sensitivity to drought is higher in young trees compared to mature trees, which means young trees activated physiological regulation processes to reduce the risks of carbon starvation and improve the adaption (Li et al., 2018). Preferential allocation of NSC to belowground root NSCs is important for plant growth and metabolism under disturbance (Montague et al., 2022). The study of O'Brien et al. (2014) demonstrated that the mortality rate of NSC-enriched tropical tree seedlings (24%) was lower than that of NSC-depleted seedlings (33%) after a 90-day period. In this study, a lower NSC concentration in old ramet indicated that the growth decline of leaves and branches with plant age caused by carbon economy under drought was more significant in mature trees than that in samplings for the reconstruction of efficient hydraulic system and carbon status (Guo et al., 2024). Therefore, the maintenance of NSC storage from aboveground organs and root systems may be related to irreversible hydraulic damage with drought duration and intensity (Kannenberg and Phillips, 2019). Fluctuations between sugar and starch are used to improve survival with the functional demands of trees under environmental stress (Kannenberg and Phillips, 2019).

### Effect of drought on SOC fractions and the relationship with aboveground carbon

SOC significantly affected the drought resistance and resilience of the forest ecosystem by microbial activities (Amarasinghe et al., 2024). Drought has been found to reduce the absolute amount of new carbon to SOC while also increasing the proportional amount according to the ^13^CO_2_ pulse labeling experiment (Wang et al., 2021b). In the present study, drought caused a significant decrease in rhizosphere SOC (*p* = 0.005), with no observed effect of ramet ages or their interaction, suggesting that roots and their activities were the important source of surface SOC under drought. These findings indicate that alterations occurring at the root–soil interface may play a crucial role in enhancing drought tolerance and resilience of SOC (Steiner et al., 2024). Extended drought in subtropical forest soils significantly transformed the composition and stability of SOC substances. The study revealed a significant reduction in POC, ROC, and MBC as well as ramet ages in response to drought, suggesting that drought resulted in decreasing the carbon allocation in plant organs to roots and rhizosphere (Wang et al., 2023b), indicating that root-derived carbon plays a more important role in soil soluble carbon (POC, ROC, and MBC) than that in MOC in rhizosphere soil under drought (Chen et al., 2016; Sun et al., 2023). Modifications occurring at the interface of roots and soil due to drought may have critical implications for the resilience of ecosystems in the context of climate change (Steiner et al., 2024).

More precisely, the relationship between SOC and the sensitivity of above-ground net primary productivity to drought exhibited a negative correlation, whereas a positive correlation was observed between SOC content and the sensitivity of belowground net primary productivity to drought (Jaman et al., 2024). In the current study, drought treatments strengthened the associations between the SC and NSC of different tissues and organs with soil carbon components on moso bamboo (**Figure 5**), suggesting that the variation in SOC during prolonged drought can be attributed to input changes in plant-derived carbon (Steiner et al., 2024). Xia et al. (2023) demonstrated that the change in soil ^13^C exhibited a decline as the plant ^13^C increased, whereas MB ^13^C content initially decreased and then increased with the rising plant ^13^C. In this study, the SC of branches exhibited positive correlations with ROC and POC, while the NSC of branches showed a positive correlation with POC, indicating the important role of branches for SOC components. This result verified that drought strengthened the linkages between branches and SOC components to meet the root growth demands or resource acquisition (Tang et al., 2022).

In this study, the SC of roots was significantly positively correlated with POC and ROC, while the NSC of roots showed a significantly positive correlation with MBC (**Figure 6**), suggesting that root-derived carbon is initially transferred to soil microbiome with enhanced resource exploitation and then sequenced within the soil. This finding is also consistent with a previous study indicating that SC and NSC could affect SOC fraction by stimulating SOC decomposition via root exudation (Adamczky et al., 2019). Additionally, the soluble carbon of POC and ROC in rhizosphere soil exhibited significant correlations with root carbon (e.g., SC), indicating that root-derived SC may increase SOC as the predominant source of SOC and that drought caused an increase in the ^13^C allocation to root and SOC fractions (Xia et al., 2023). Our findings highlighted that plant–soil carbon feedback in clonal plants influences soil carbon cycling, which would help to understand the group drought resistance strategy of clonal plants.

## Conclusions

Carbon input through plant organs into the soil is a major flux in the global carbon cycle and is crucial for carbon sequestration and ecosystem stability under climate change. In this study, we aimed to examine the interaction between organ carbon and SOC fraction among different-aged ramets on moso bamboo under drought conditions. The study indicated that drought-induced plasticity of organ chemical traits can affect soil organic carbon storage with a more resource-conservative strategy. It also highlighted the alteration in carbon allocation has consequences for soil carbon storage during longer-term drought, where leaves function as potent carbon sinks, leading to increased accumulation of soluble sugars and starch, as drought intensifies. The soil storage depends strongly on moso bamboo age and organ carbon. These results propose that the ramet plant prioritizes the investment of assimilates belowground (root) for regaining root function during drought. The study indicates that the interlinked organ carbon with soil organic carbon stability is hampered by the response of these processes with species, ramet age, and drought intensity. Such linkages of plant-derived carbon and SOC stability will improve integrative ecological understanding of ecosystem carbon cycling processes under drought.

## Data Availability

The original contributions presented in the study are included in the article/s **Supplementary Material**. Further inquiries can be directed to the corresponding author.
