# Supplementary material for: Drought induces opposite changes in organ carbon and soil organic carbon to increase resistance on moso bamboo
Source: Front Plant Sci. 2024 Nov 26;15:1474671. doi: 10.3389/fpls.2024.1474671 (PMC11628292; doi:10.3389/fpls.2024.1474671)
Supplement: Supplementary file 1 [file DataSheet1.pdf]

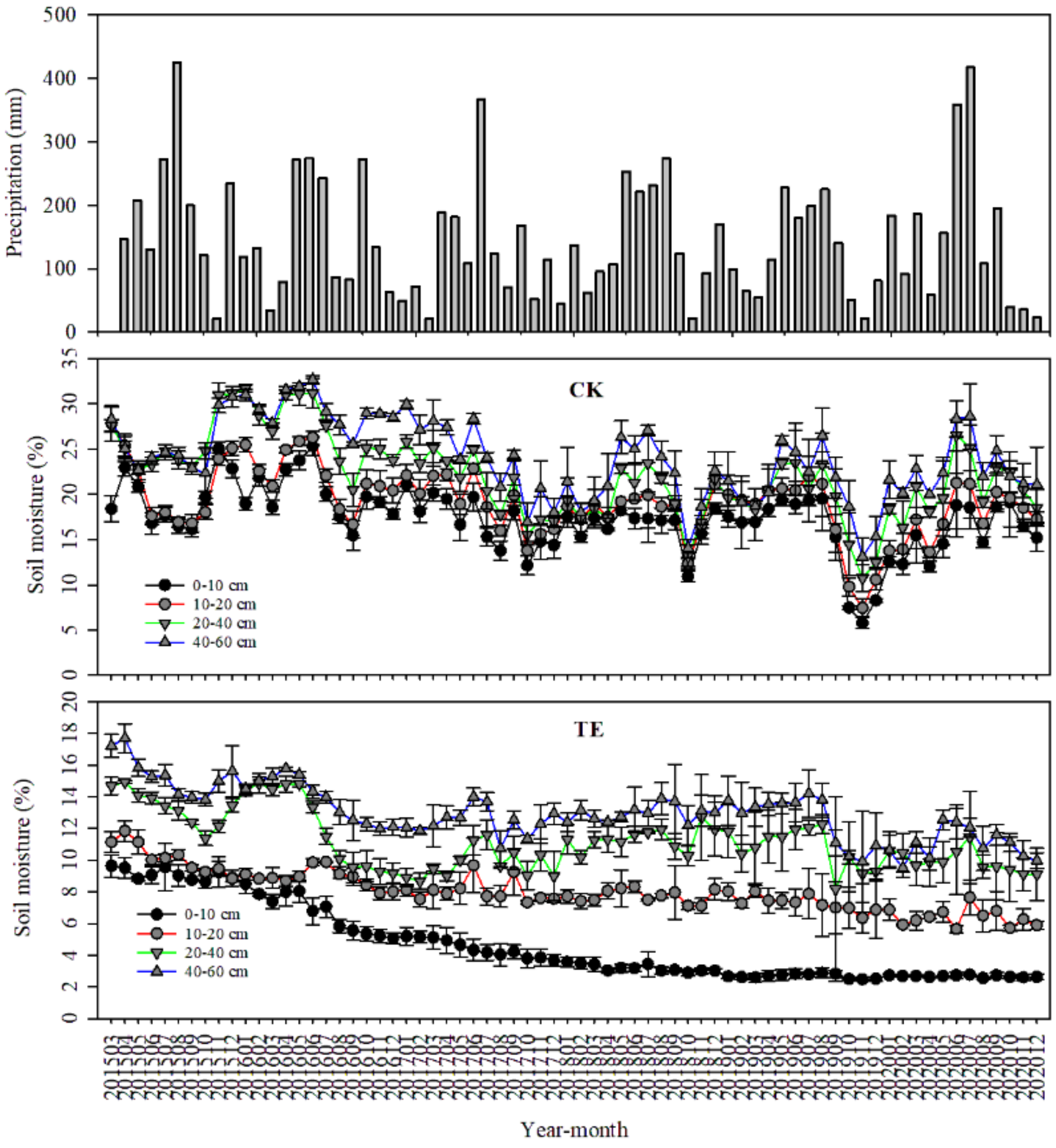

Figure 1S Precipitation and soil water moisture (0–60 cm) in drought-stressed plots (TE) and ambient controls (CK) across the drought-treatment experiment period in moso bamboo stands.
